# Benefit-Cost Analysis of a Package of Early Childhood Interventions to Improve Nutrition in Haiti^1^

**DOI:** 10.1017/bca.2019.1

**Published:** 2019-02-28

**Authors:** Brad Wong, Mark Radin

**Affiliations:** 1 Copenhagen Consensus Center, USA; 2 Department of Environmental Sciences & Engineering, University of North Carolina at Chapel Hill, Chapel Hill, NC 27599, USA

**Keywords:** education and human capital, health, international

## Abstract

We conduct a benefit-cost analysis of a package of early childhood interventions that can improve nutrition outcomes in Haiti. Using the Lives Saved Tool, we expect that this package can prevent approximately 55,000 cases of child stunting, 7,600 low-weight births and 28,000 cases of maternal anemia annually, if coverage reaches 90% of the target population. In addition, we expect these nutrition improvements will avoid 1,830 under-five deaths, 80 maternal deaths and 900,000 episodes of child illness every year. Those who avoid stunting will experience lifetime productivity benefits equivalent to five times gross national income per capita in present value terms, at a 5% discount rate. While previous benefit-cost analyses of this specific package have only estimated the lifetime productivity benefits of avoided stunting, this paper also accounts for reductions in fatal and non-fatal health risks. In the base case scenario, the annualized net benefits of the intervention equal Haitian gourdes 13.4 billion (USD 211 million) and the benefit-cost ratio (BCR) is 5.2. Despite these substantial benefits, the package may not be the most efficient use of a marginal dollar, with alternative interventions to improve human capital yielding BCRs approximately three to four times higher than the base estimate.

**JEL classifications:** O2; I1; I2.

## 1 Introduction

Haiti has the poorest health and education outcomes in the Western Hemisphere. Its infant mortality rate (51 deaths per 1000 live births in 2016) is three times higher than the Latin America and Caribbean average, while the maternal mortality rate (359 per 100,000 live births) is five times higher than the regional benchmark (World Bank, 2017). In terms of education, the adult literacy rate in 2015 was 61% while the Caribbean average was 92% (Central Intelligence Agency Factbook, 2018). For a country hunting for sources of economic growth, the future economic development of the country will require a significantly improved human capital base.

At the same time, the available resources to solve Haiti’s myriad problems are not particularly large. In 2016, government and donors spent roughly Haitian gourdes (HTG) 180 billion or USD 3 billion (Central Intelligence Agency Fact Book, 2016; AidFlows, 2017) equaling HTG 17,000 or USD 270 per capita on providing services to the Haitian people. Benefit-cost analysis is a useful tool to determine effective uses of Haiti’s limited funds. It is against this backdrop that this paper undertakes a benefit-cost analysis of an intervention that addresses both health and education related challenges in Haiti, and has the potential to be highly effective: the provision of a package of early childhood interventions to address nutrition-related outcomes such as stunting, wasting and low birthweight. An analysis of this package is motivated by the fact that the intervention has been assessed as one of the most effective uses of development funds globally^2^ but a benefit-cost analysis has not been previously undertaken in the Haitian context.

This paper also extends the literature on the benefit-cost analysis of this particular set of interventions by accounting for both health benefits, which in this paper we define as reductions in fatal and non-fatal health risks,^3^ and improved lifetime productivity from avoided stunting. Previous analyses have only accounted for the latter (Hoddinott et al., 2013*a*,*b*; Horton & Hoddinott, 2014). This is of more than academic interest. The results of previous analyses appear to have had a significant impact on policy, for example, being cited in outcome documents that committed USD 4.15 billion toward implementing elements of this package of interventions (Global Nutrition for Growth Compact, 2013). Omitting the benefits associated with reductions in mortality and non-fatal health risks understates the effectiveness of this intervention and may have implications for future nutrition funding. We find health impacts represent 13–58% of the total benefits depending on the discount rate applied, so their inclusion is material.^4^

Additionally, because the nutrition intervention leads to three types of benefits – decreased mortality risks, avoided non-fatal health risks and increased lifetime productivity – reflecting both immediate and longer-term impacts, we are able to test the effects of numerous valuation and discounting assumptions on the results (Robinson & Hammitt, 2018; Robinson et al., 2019).

This paper uses the Lives Saved Tool commonly shortened to LiST (see https://www.livessavedtool.org/ for further details) to model both the nutrition and health effects of scaling up this package of interventions to cover 90% of pregnant women and 0–2-year-old children in Haiti. This intervention is expected to prevent approximately 55,000 cases of child stunting, 7,600 babies being born with low birthweight and 28,000 cases of maternal anemia. These improvements in nutritional status are expected to prevent 1,830 under-five deaths, 80 maternal deaths and 900,000 episodes of child illness every year. For those children who avoid stunting, it is expected to also deliver wage benefits equivalent to five times gross national income (GNI) per capita in present value terms, at a 5% discount rate.

In the base case scenario, our results indicate that the provision of this package is an effective use of development money, with annualized net benefits of HTG 13.4 billion or USD 211 million per year^5^ and a benefit-cost ratio (BCR) of 5.2. Seventy-seven percent of the benefits are due to lifetime productivity improvements, 19% are from avoided mortality risks, and the remainder is avoided non-fatal health risks.^6^ Despite these impressive benefits, the package may not be the most effective nutrition or education intervention in the Haitian context in terms of net benefits or the BCR. A recently completed set of 85 benefit-cost analyses for Haiti demonstrates several nutrition and education interventions that have higher BCRs, some significantly so (Lomborg et al., 2018). For example, the central BCR estimate for wheat flour fortification is 24 (Engle-Stone et al., 2017), while for early childhood stimulation it is 17 (Rabbani, 2017). In terms of improving human capital, the nutrition package may not be the most effective use of a marginal dollar (or gourde), even after accounting for uncertainty in the results.

Additional analyses indicate that the results are sensitive to the choice of valuation approaches adopted. In this study, the value of mortality and non-fatal health risk reductions are assessed using recommendations from Robinson et al. (2019) and Robinson and Hammitt (2018) respectively. At the 5% real discount rate, adopting the valuation methods with the largest unit values leads to net benefits of HTG 25.4 billion (USD 401 million) per year, while adopting the valuation methods with smallest unit values results in net benefits of HTG 11.8 billion (USD 186 million). The range from the lower bound to the upper bound estimate is HTG 13.6 billion (USD 215 million), which is larger than our base case estimate. The net benefits range from HTG 3 billion to HTG 24 billion (USD 48 million to USD 374 million) using a 12% and 3% discount rate respectively. The net benefits range from HTG 5.3 billion to HTG 22.6 billion (USD 84 million to USD 357 million) due to variation in the impact of the intervention package on health and nutrition outcomes. Finally, the net benefits range from HTG 4.6 billion to HTG 18.6 billion (USD 73 million to USD 294 million) due to variation in the estimates on the impact of stunting on lifetime productivity.

## 2 Haiti’s nutrition challenges

Haiti’s most recent demographic and health survey, Enquête Mortalité, Morbidité et Utilisation des Services (EMMUS-VI) 2016–17 (IHE and ICF, 2017), shows that the country continues to face significant nutrition challenges, and is likely off-track on four out of the six World Health Assembly 2025 Global Nutrition Targets. Comparing EMMUS-VI 2016–17 with the prior demographic and health survey, EMMUS-V 2012 (Ministry of Public Health and Population, 2013), the nutrition landscape appears virtually unchanged over five years across a range of key indicators such as stunting, anemia and exclusive breastfeeding (see Table 1). Undernutrition is a source of concern because it impacts both health outcomes (Fishman et al., 2004; Black et al., 2008; Olofin et al., 2013), as well as current and future productivity (Haas & Brownlie, 2001; Alderman & Behrman, 2006; Prendergast & Humphrey, 2014).

**Table 1:** Haiti’s progress on 2025 Global Nutrition Targets.

WHA 2025 Global Nutrition Targets	2012 Baseline and 2025 Target	Current status	Status
1. Stunting: 40% reduction in the number of children under 5 who are stunted	Baseline: 21.9%^*^ Target: 13.6%	2016: 21.9%^**^	Off-track
2. Anemia: 50% reduction in anemia of women of reproductive age	Baseline: 46.0%^***^ Target: 23.0%	2016: 48.8%^**^	Off-track
3. Low birthweight: 30% reduction in low birthweight	Baseline: 18–23%^****^ Target: 13–16%	2016: No data	Unknown but likely off-track
4. Childhood overweight: No increase in childhood overweight	Baseline: 3.6%^*^ Target: 3.6%	2016: 3.4%^**^	On-track
5. Breastfeeding: Increase the rate of exclusive breastfeeding in the first 6 months up to at least 50%	Baseline: 39.7%^*^ Target: 50.0%	2016: 39.9%^**^	Off-track
6. Wasting: Reduce and maintain childhood wasting to less than 5%	Baseline: 5.2%^*^ Target: 5.0%	2016: 3.7%^**^	On-track

* From EMMUS-V.

** From EMMUS-VI.

*** Micronutrients database, WHO (2014).

**** Lower bound from LiST based on approach in Kozuki et al., (2017), upper bound from UNICEFlow birthweight database adapted from EMMUS-V.

The most recent data from 2016 indicates that 22% of Haitian children under five years old are stunted (IHE and ICF, 2017). This compares to 7% in neighboring Dominican Republic in 2013 and 11% in the Latin American and Caribbean region in 2014 (World Bank, 2017). Anemia among women of reproductive age continues to be a major problem in Haiti with the most recent survey data indicating anemia prevalence of 49%, the highest rate among Latin American and Caribbean countries (Mujica-Coopman et al., 2015). Low birthweight is likely to be a significant challenge for Haiti, though ascertaining accurate data on the current rate is difficult. There is no recognized, widely accepted time series data for low birth weight globally that would allow accurate tracking against World Health Assembly targets (World Health Organization, 2017). Using EMMUS-V, WHO estimates that 23% of children were born with low birth weight (UNICEF, 2014). LiST recently introduced a mechanism to estimate low birthweight and notes a 2012 rate of 18% for Haiti. (Kozuki et al., 2017). Given that stunting is one major consequence of sub-optimal birth outcomes (LiST, 2016; Rahman et al., 2016; Aryastami et al., 2017), and the prevalence of stunting has not changed in five years, it is highly possible there have been no improvements in the low-birthweight indicator since 2012. Exclusive breastfeeding in infants 0–5 months has remained basically unchanged between 2012 and 2016, with the rate plateauing at 40%. Two aspects of nutrition appear to be on-track in Haiti. Prevalence of wasting has shown steady decline over the last decade, reducing from 10.1% in 2005–06 (EMMUS-IV) to 5% in EMMUS-V and then to 3.7% in 2016–17 (EMMUS-VI). Child overweight remains below the World Health Assembly target of 5%.

## 3 Intervention description and LiST approach

Bhutta et al. (2013) describe a package of ten evidence-based interventions to address various forms of undernutrition, some elements targeted at pregnant women, others targeted at children aged 0–2 and one, salt iodization, targeted at the entire population. In this paper, the focus is on the entire package because together they represent a series of nutritional interventions that have gathered international public health interest^7^ and have already been widely studied in terms of costs, impact and cost-effectiveness (Bhutta et al., 2013; Hoddinott et al., 2013*a*,*b*). It is possible in the Haitian context that individual components or alternative combinations of these ten interventions would have higher BCRs, but identifying the optimal set is beyond the scope of this paper.

Using a previous version of LiST, Bhutta et al. estimate that scaling up this package of interventions to 90% coverage rates of the target population in 34 countries will help reduce stunting by 20% as well as avoid 1,000,000 under-five deaths. The interventions, target population and total cost in 2010 Int$ are summarized in Table 2.

**Table 2:** Package of interventions to improve maternal and child nutrition, target and beneficiary populations, and estimated costs to scale up coverage to 90% in 34 countries.

Intervention	Intervention population	Beneficiary population	Cost (in 2010 Int$, million)
Salt iodization	Whole population	Whole population	$68
Multiple micronutrient supplementation in pregnancy, including iron folate	Pregnant women	Pregnant women and children in utero	$472
Calcium supplementation in pregnancy	Pregnant women	Pregnant women and children in utero	$1,914
Energy protein supplementation in pregnancy	Pregnant women	Pregnant women and children in utero	$972
Vitamin A supplementation in childhood	Children 6–59 months	Children 6–59 months	$106
Zinc supplementation in childhood	Children 12–59 months	Children 12–59 months	$1,182
Breastfeeding promotion	Pregnant women mothers of children aged 0–5 months	Children aged 0–6 months	$653
Complementary feeding education	Mothers of children aged 6–23 months	Children aged 6–23 months	$269
Complementary food supplementation	Mothers of children aged 6–23 months	Children aged 6–23 months	$1,359
SAM Management	Children 6–23 months severely wasted	Children aged 6–23 months	$2,563
TOTAL			$9,559

Source: Adapted from Bhutta et al. (2013).

After the 2013 *Lancet* Nutrition Series, further updates to LiST were made in 2015 to reflect the latest and best scientific evidence (Clermont & Walker, 2017). LiST models both the direct effects of interventions on fatal and non-fatal health risks, as well as the indirect effect, via nutrition pathways on health outcomes. For example, the effect of complementary feeding education is first modeled as a 30% reduction in the odds of stunting following Panjwani and Heidkamp (2017). The software then models the subsequent reduction in stunting on mortality outcomes using the relative risk associations between stunting and child mortality from Olofin et al. (2013). In this way, the model is able to produce estimates of key nutrition outcomes as well as avoided deaths from the interventions. Table 3 below outlines the interventions modeled in LiST for this paper, including the presumed baseline coverage of these interventions in Haiti.

**Table 3:** LiST inputs.

LiST intervention category	LiST intervention name	Baseline coverage	Source of intervention baseline coverage	Intervention coverage
Pregnancy	Multiple micronutrient supplementation in pregnancy	0%	Assumed based on Engle-Stone et al. (2017)	90%
Pregnancy	Calcium supplementation	0%	Assumed based on Engle-Stone et al. (2017)	90%
Pregnancy	Balanced energy supplementation	0%	Assumed based on Engle-Stone et al. (2017)	90%
Breastfeeding	Promotion of breastfeeding (Health System AND Home / Community)	36.7%	LiST proxy: % of those exclusively breastfeeding from EMMUS-VI	90%
Preventative	Complementary feeding – education only	29.3%	LiST proxy: Those living on less than $1.90 per day sourced from EMMUS-VI	90%
Preventative	Complementary feeding – supplementation and education	29.3%	LiST proxy: Those living on less than $1.90 per day sourced from EMMUS-VI	90%
Preventative	Vitamin A supplementation	30%	UNICEF Vitamin A coverage database	90%
Preventative	Zinc supplementation	0%	Assumed	90%
Curative	SAM treatment for severe acute malnutrition	80%	Vosti and Adams (2017)	90%

It is difficult to precisely identify coverage rates for the interventions in Haiti. Where possible, we infer coverage rates from existing studies and if these were unavailable, use proxies applied by LiST. Pregnancy-related interventions are from Engle-Stone et al. (2017), which supposes no coverage of the pregnancy interventions, assuming that instead women are receiving only iron and folic acid tablets. Proxies embedded in LiST have been applied for breastfeeding promotion, complementary feeding (education) and complementary feeding (education and supplementation). Vitamin A supplementation is from a UNICEF Vitamin A coverage database and is also a LiST default. Last, severe acute malnutrition treatment coverage is assumed to be 80%, following Vosti and Adams (2017).

The following provides a short overview of some of the modeled effects and latest evidence behind the interventions, although interested readers should consult the 2017 *Journal of Nutrition* supplement on nutrition modeling in LiST for more detailed assessments of the evidence including limitations (Walker and Clermont, 2017) as well as the LiST visualizer tool (http://listvisualizer.org/).

The three pregnancy-related interventions directly impact maternal mortality, maternal anemia, and birth outcomes, with flow-on effects to other nutrition-related outcomes and child mortality. Calcium supplementation is the only intervention that affects maternal mortality directly, resulting in avoided pre-eclampsia and a 20% lower mortality risk for food-insecure mothers (Ronsmans & Campbell, 2011). The intervention also reduces preterm births by 12% (Imdad et al., 2011*a*). Multiple micronutrient supplementation lowers maternal anemia by 67% (Pñna-Rosas et al., 2015). Multiple micronutrient supplementation also reduces the risk of preterm birth by 6% or 16% depending on the mother’s body mass index at birth, and the risk of small for gestational age births by 3% for women with a body mass index greater than 18.5 (Smith et al., 2017). The provision of balanced energy protein to food-insecure pregnant women leads to a 21% reduction in births that are small for gestational age (Ota et al., 2015).

Three interventions have a direct impact on stunting with effect sizes dependent on the food security status of the beneficiary household. Relative to food-secure households exposed to complementary feeding promotion, the odds of stunting are 1.3 times higher for food-secure populations without promotion, 1.74 times higher for food-insecure households with promotion and supplementation, and 1.95 times higher for food-insecure populations without any intervention (Panjwani & Heidkamp, 2017). Zinc supplementation leads to a 10% reduction in the odds of stunting (Bhutta et al., 2013), while also reducing mortality from pneumonia and diarrhea for zinc-deficient populations (Yakoob et al., 2011). Reductions in stunting are translated to reductions in disease-specific child mortality using relative risks from Olofin et al. (2013).

The remaining interventions affect child mortality and disease. Vitamin A supplementation reduces diarrhea-related mortality by 53% and the incidence of diarrhea by 38% for vitamin-A-deficient children (Imdad et al., 2011*b*). Promotion of breastfeeding leads to an increase in early, exclusive and extended breastfeeding, with effect sizes dependent on the location of promotion (Sinha et al., 2017), Sub-optimal breastfeeding increases mortality risk by 1.35 to 5.4 times relative to optimal breastfeeding depending on the age of the child and cause (Lamberti et al., 2013; NEOVITA, 2016; LiST, 2017). Treatment for severe acute malnutrition increases recovery rate from wasting by 78%, while being wasted leads to an increase in cause-specific mortality risk between 1 and 12 times depending on the severity of wasting (Olofin et al., 2013).

## 4 Nutrition and health impacts from the intervention package

The time period chosen for the analysis is 2016–2025. The year 2016 corresponds to the most recent year for which GNI per capita data is available from the World Bank database (World Bank, 2018). The end-point corresponds to the final year of the World Health Assembly Global Nutrition Targets. Each year, the intervention will cover 90% of all pregnant women and 0–2-year-old children. The average number of births over the ten years is 260,000 annually according to DemProject, a population projection package that comes with LiST.

The intervention package leads to a wide array of improved nutrition outcomes. The intervention reduces the prevalence of stunting by 20% (95% C.I. 11.7%–30.7%), the same effect size as documented for 34 countries in Bhutta et al. (2013). It takes four years for the full effects to materialize; but by 2020, in expectation the intervention package avoids 55,000 cases of stunting every year. This would reduce the prevalence of stunting from 22% to 17.5%, approximately halfway to meeting Global Nutrition Target 1.

Additionally, 28,000 cases of maternal anemia would be avoided per year due to multiple micronutrient supplementation. This would reduce the prevalence of maternal anemia by 23%. Given the focus of the intervention on pregnant women, it does not have a significant impact on Global Nutrition Target 2, which covers all women of reproductive age.

The intervention would improve birth outcomes significantly. LiST estimates that the prevalence of low birthweight would fall from 18% to 15%, a reduction of 16%. This corresponds to roughly 7,600 low-birthweight births avoided per year and represents a halfway movement toward Global Nutrition Target 3. Rates of exclusive breastfeeding due to promotion in health system, homes and communities would increase from 42% to 62%, enabling Haiti to fully meet Global Nutrition Target 4.

Due to direct and indirect mechanisms, LiST predicts that the package of interventions would avoid, on average, 1,826 under-five deaths (including 577 neonatal deaths) and 82 maternal deaths for a total of 1,908 avoided premature deaths per year. For children, almost 90% of the deaths avoided are from diarrheal and respiratory causes. The package would reduce the child mortality rate by 10% to 61 per 1,000, the neonatal mortality rate by 9% to 23 per 1,000 and the maternal mortality rate by 9% to 328 per 100,000 live births. Results are summarized below in Table 4.

**Table 4:** Average annual number of child, neonatal and maternal deaths avoided due to intervention.

Causes	Neonatal deaths avoided (*<*1 month)	Non-neonatal child deaths avoided (1–59 months)	Maternal deaths avoided	Total
Pneumonia	55 (95% CI: 12, 94)	645 (95% CI: 58, 1,270)	—	700 (95% CI: 70, 1,364)
Diarrhea	2 (95% CI: 1, 3)	416 (95% CI: 123, 663)	—	418 (95% CI: 124, 666)
Prematurity	184 (95% CI: 25, 326)	—	—	184 (95% CI: 25, 326)
Asphyxia	177 (95% CI: 29, 315)	—	—	177 (95% CI: 29, 315)
Sepsis	159 (95% CI: 35, 268)	—	—	159 (95% CI: 35, 268)
Other causes	—	188 (95% CI: 73, 384)	82 (95% CI: 25, 124)	270 (95% CI: 98, 508)
All	577 (95% CI: 101, 1,005)	1,249 (95% CI: 254, 2,317)	82 (95% CI: 25, 124)	1,908 (95% CI: 355, 3,323)

LiST also predicts that on average, the interventions would avoid 893,000 (95% C.I. 529,000–1,226,000) cases of diarrhea annually.

## 5 Benefit-cost analysis

### 5.1 Common assumptions – assumed growth and discounting

Estimating the value of future mortality risk reductions, non-fatal health risk reductions and productivity increases requires forecasting GNI per capita growth, given that these benefits are expected to increase with income. The International Monetary Fund provides forecasts of short-term per capita growth rates for Haiti of approximately 2.5% over the years 2016–2020, which we also apply to this study (IMF, 2015). Haiti is infamous for having extremely poor per capita output growth throughout the last century, with periods of significant real output decline (Khan, 2010). In purchasing power parity (PPP) terms, per capita GNI grew only 0.6% per annum over the period 1970 to 2015 (Penn World Tables – Feenstra et al., 2015). Therefore, from 2020 we assume that growth rates decrease linearly from 2.5% to 0.6% over 20 years, and beyond 2039 the long-term growth rate is 0.6% per annum.^8^

This paper uses discount rates of 3%, 5% and 12%. Three percent is used following recommendations of the iDSI reference case (Wilkinson et al., 2016) and also since it is a common value adopted in health economics papers. Five percent is twice the near-term growth rate, and motivated by the Ramsey rule (Claxton et al., 2019). Additionally, 5% is applied in other benefit-cost analyses of this intervention including those commissioned by Copenhagen Consensus (Hoddinott et al., 2013*b*; Horton & Hoddinott, 2014). The use of a 12% discount rate is based on the advice of a council of senior Haitian economists who suggested this value as an appropriate social discount rate for the country. This council had gathered for the *Haiti Priorise* ^9^ exercise conducted by the Copenhagen Consensus Center.

### 5.2 Costs

In previous analyses, the cost of this intervention for a range of developing countries has been estimated at around Int$ 100 per child for a two-year program in 2010 Int$ (Bhutta et al., 2013; Hoddinott et al., 2013*a*; Horton & Hoddinott, 2014). Several factors such as poor infrastructure, a fragmented health landscape consisting of private, public and NGO facilities working in isolation and limited government administrative capacity, mean that costs are likely to be higher for Haiti.

Studies in the *Haiti Priorise* series are used to calibrate costs of this package. Engle-Stone et al. (2017) perform a benefit-cost analysis of micronutrient provision to pregnant women and find a cost of HTG 2680 (USD 42) to deliver micronutrients *and* calcium supplementation to 90% of pregnant women via community health workers. That paper also identifies a cost of HTG 160 (USD 2.5) per child provided with multiple micronutrients, including Vitamin A. Costs in that paper were estimated using an “ingredients approach”: identifying and modeling the individual components – transport, packaging, storage, supplements, health workers to deliver the interventions at scale – and verifying costs with organizations working “on-the-ground” in Haiti.

This paper uses these costs for the elements in the intervention package that are the same with two important adjustments. First, we adjust for diseconomies of scale as per Vosti and Adams (2017). As coverage increases, it is likely that community health workers would need to reach more remote and inaccessible villages, taking more travel time and seeing fewer individuals. Vosti and Adams (2017) develop a graduated scale of this effect for Haiti and estimate that reaching 90% of the population requires approximately 20% more cost per person than reaching just half. Therefore, we apply a 20% markup to unit costs presented in Engle-Stone et al. (2017) since it appears that adjustments for scale were not made in that paper. This results in a cost of around HTG 3,200 (USD 50) per pregnant women provided with multiple micronutrients and calcium supplementation.

Second, the HTG 160 (USD 2.5) unit cost of providing micronutrients to children from Engle-Stone et al. (2017) is for a program that reaches 50% of children as they naturally interact with the health system during vaccinations. Since the proposed coverage of the package we assess is 90%, additional community health workers are required to reach the last 40% via an outreach program. For a cohort size of 260,000 and an annual health worker load of 1,500 home visits annually, we estimate that scaling up to reach 90% of children would require 168 extra community health workers per year, including adjustments for scale mentioned above. Assuming salaries of HTG 60,000 (USD 950) per year, this would increase unit costs of this part of the package to around HTG 250 (USD 4) per child.

There are no Haiti-specific costs for other parts of the package, so we use these two unit cost figures as reference points with which to estimate the intervention cost in the Haitian context. Specifically, we assume that the costs of the unknown components are *proportionally* the same with respect to the known components as they were originally presented in Bhutta et al.^10^ The HTG 3,200 (USD 50) cost to deliver micronutrients and calcium supplementation in pregnancy is the reference point for the interventions delivered to mothers, and the cost of HTG 250 (USD 4) to deliver multiple micronutrients is the reference point for interventions targeted at children.

For example, in Bhutta et al. (2013), the per mother cost of breastfeeding promotion is 57% the cost of delivering micronutrients and calcium supplementation. Therefore, knowing that micronutrients and calcium cost HTG 3,200 (USD 50) per mother, the cost of breastfeeding promotion is calculated as 57%*HTG 3,200 (USD 50) = HTG 1,830 (USD 30) per pregnant woman. The same approach is applied to components directed at *children* using the HTG 250 (USD 4) for micronutrient supplementation as the reference unit cost for Vitamin A supplementation.

Community management of severe acute malnutrition and salt iodization represent unique cases that need to be considered differently. Following Hoddinott et al. (2013*a*), the unit costs per beneficiary of treating severe acute malnutrition are calculated by multiplying the cost of treatment, estimated at HTG 6,334 (USD 100) per wasted child (Vosti & Adams, 2017), by twice the prevalence rate of wasting. This leads to a unit cost of HTG 165 (USD 3) for each child receiving the intervention.^11^ Salt iodization is assumed to cost HTG 2 (USD .03) per child treated, the cost identified in Hoddinott et al. (2013*a*), adjusted for inflation and converted to gourdes at market rates.

The estimated unit costs for the intervention components are presented in Table 5. The total cost of the package is HTG 16,222 per child, or USD 248. To reach scale, this intervention would require an additional HTG 3.0 billion or USD 48 million in the first year, 2016. In subsequent years, we assume that half the costs of the package are attributable to labor which increase at the same rate as GNI per capita growth rate.

**Table 5:** Estimated costs per child and costs to scale up to 90% coverage in the first year.

Intervention	Estimated unit cost for Haiti, 2016 (in HTG and USD)	Estimated annual costs to scale to 90% (in HTG and USD)	Basis for unit cost estimation
Salt iodization	HTG 2.00 USD 0.03	HTG 0.5 m USD 0.01 m	Following Hoddinott et al. (2013*a*) adjusted for inflation
Multiple micronutrient supplementation in pregnancy, including iron folate	HTG 3,200 USD 42.00	HTG 746 m USD 11.8 m	Estimated in Engle-Stone et al. (2017)
Calcium supplementation in pregnancy	Incl. in above	Incl. in above	Estimated in Engle-Stone et al. (2017)
Energy protein supplementation in pregnancy	HTG 3,195 USD 50.44	HTG 745 m USD 11.8 m	Proportional adjustment based on 2660 HTG to deliver MMN and Ca to pregnant women in Haiti
Vitamin A supplementation in childhood	HTG 250 USD 3.95	HTG 39 m USD 0.6 m	Estimated in Engle-Stone et al. (2017)
Zinc supplementation in childhood	HTG 518 USD 8.17	HTG 121 m USD 1.9 m	Proportional adjustment based on HTG 320 to deliver Vitamin A to children in Haiti
Breastfeeding promotion	HTG 1,830 USD 28.89	HTG 253 m USD 4.0 m	Proportional adjustment based on HTG 2660 to deliver MMN and Ca to pregnant women in Haiti
Complementary feeding education	HTG 673 USD 10.63	HTG 106 m USD 1.7 m	Proportional adjustment based on HTG 2660 to deliver MMN and Ca to pregnant women in Haiti
Complementary food supplementation	HTG 6,390 USD 100.88	HTG 1,005 m USD 15.9 m	Proportional adjustment based on HTG 2660 to deliver MMN and Ca to pregnant women in Haiti
SAM Management	HTG 165 USD 2.60	HTG 4.3 m USD 0.1 m	HTG 6344 per child treated estimated in Vosti and Adams (2017) multiplied by twice prevalence rate as per Hoddinott et al., 2013a
**TOTAL**	HTG 16,222 USD 248	HTG 3,019 m USD 47.7 m	

The scope of this intervention package – 10 concurrent nutrition interventions – would be unprecedented in Haiti. However, it is likely that implementing it together as a package would be less costly than the sum of separate decentralized programs. One of the well-known issues in Haiti’s development landscape is sub-optimal levels of coordination between governments and international aid groups (Ramachandran & Walz, 2015; Kligerman et al., 2017). Implementing the package using centrally coordinated operations, ideally with Haitian government involvement, would not only allow for some savings in program costs, but also limit the possibility of different aid groups acting at cross purposes.

### 5.3 Benefits from avoided premature mortality

Section 4 indicates that the intervention package reduces mortality risk, leading to 1908 avoided deaths per year. Ideally, one would value these risk reductions using stated or revealed preference studies that estimate individual willingness to pay (WTP) among the beneficiary population in Haiti. To the best of our knowledge there are no such studies. The review by Robinson et al. (2019) also did not identify any Haiti-specific literature. Instead, avoided mortality benefits are valued using the alternative approaches suggested by Robinson et al. (2019). These are:

(i) Estimate a value of statistical life (VSL)^12^ by transferring a value derived from the US (USD 9.4 million in 2016 US dollars, equivalent to 160× GNI per capita PPP) using an income elasticity of 1.5. This corresponds to an initial value of HTG 1.3 m (Int$ 51,000 or USD 20,000) representing a multiplier of 28× GNI per capita PPP. This value grows with income according to the relationship $VSL_{t}=VSL_{0}*{\left[\Pi_{0}^{t}\left(1+g_{t}\right)\right]}^{1.5}$ where *g_t_* is the growth rate in GNI per capita in period *t*, described in Section 5.1.
(ii) Estimate a constant value of statistical life year (VSLY) by dividing the population-average VSL for each year from the approach above by the undiscounted life expectancy of the average Haitian adult (36 years). The VSLY is therefore HTG 36,000 (USD 500) in 2016, growing proportionally with VSL growth noted above. According to Haiti life tables, an avoided child death at age two yields 66 avoided years of life lost (YLL), and an avoided maternal death at age 27 yields 46 avoided YLLs. These YLLs are multiplied by deaths avoided and the VSLY to estimate mortality benefits.
(iii) Robinson et al. (2019) suggest alternative VSLs of 100× and 160× GNI per capita PPP, using base values from the OECD and U.S. respectively and assuming an income elasticity of 1.0. These imply a VSL of HTG 4.5 million (USD 71,000) and HTG 7.2 million (USD 113,000) respectively.

The results of each of these approaches to valuation are presented in Table 6.

**Table 6:** Annualized benefit from avoided mortality.

Discount rate	Base Case: Deaths avoided valued at 1.3 m HTG VSL	Alternative 1: Avoided YLLs valued at 36,000 HTG VSLY	Alternative 2: Deaths avoided valued at 4.5 m HTG VSL	Alternative 3: Deaths avoided valued at 7.2 m HTG VSL
3%	HTG 3,129 USD 49	HTG 5,802 USD 92	HTG 9,493 USD 150	HTG 15,189 USD 240
5%	HTG 3,101 USD 49	HTG 5,747 USD 91	HTG 9,459 USD 149	HTG 15,135 USD 239
12%	HTG 3,009 USD 47	HTG 5,566 USD 88	HTG 9,348 USD 148	HTG 14,957 USD 236

Source: Estimates by the authors. All values are in millions of HTG or millions of USD.

The results indicate that the choice of estimates has a large effect on the results. In the base case the annualized value of avoided mortality is HTG 2.8 billion (USD 45 million) at a 5% discount rate. Moving from a VSL to a VSLY approach has a significant upward effect on the benefits calculation, since children and mothers are younger than the average Haitian adult. This approach results in an annualized mortality benefit value of HTG 5.3 billion (USD 83 million), 85% higher than the base case. Alternative VSL estimates suggested by Robinson et al. (2019) – i.e. GNI per capita multipliers of 100 and 160 – lead to mortality benefits more than 3× and 5× the base case respectively. The choice of discount rate has minimal effect on the value of avoided premature mortality.

### 5.4 Benefits from reduced non-fatal health risks

Benefits from reduced non-fatal health risks are valued using two approaches suggested by Robinson and Hammitt (2018), namely (i) using WTP estimates and (ii) valuing years lost to disability (YLD) at a constant VSLY.^13^ Generally, WTP estimates are preferred, if they are of sufficient quality and applicable to the population and risks of concern. We use both approaches due to concerns about the suitability of the WTP study. In both cases, third-party costs can be added.

For the first approach, we searched the available literature for high-quality studies that estimate individual WTP for avoiding diarrheal disease in developing countries. We focus on diarrhea because this makes up the vast majority of the benefits gained from reduced non-fatal health risks. The only relevant study is Guh et al. (2008), in which the authors survey respondents from rural China to estimate their WTP to avoid cases of shigellosis, a leading cause of diarrhea. Based on the responses of caregivers, individual WTP to avoid a case of diarrhea in children under five was Int$ 35.40 (2002 international dollars)^14^ which is 1.6% of annual per capita income of respondents. This is transferred to the Haitian context using GNI per capita PPP and an income elasticity of 1.0 for a value of Int$ 29, HTG 715 or USD 11.30 per case of diarrhea in 2016, growing each year with assumed real income growth.

Additionally, Robinson and Hammitt (2018) suggest an alternative approach where each YLD is valued at a constant VSLY + costs borne by third parties. The approach for estimating VSLY is discussed in the previous section with a value of HTG 32,000 (USD 500) in 2016. Each incidence of diarrhea corresponds to 0.0019 YLDs (Global Burden of Disease, 2016). Three types of costs borne by third parties are calculated: (i) outpatient costs (ii) inpatient costs and (iii) cost of caregiver time. In Haiti, there is a mix of private, non-governmental organization and public health care facilities, so some portion of costs are borne by external parties. The cost of caregiver time is included because estimates of VSL and VSLY are based on WTP to reduce risks to one’s self, and do not include the costs borne by others. The total value of the monetized YLD + third-party costs is $Int 12, HTG 306 and USD 4.8 per case of diarrhea in 2016^15^ (see appendix for assumptions used in calculation).

The results of the two valuation approaches are presented in Table 7.

**Table 7:** Annualized benefit from avoided non-fatal health risks.

Discount rate	Base Case: Benefit transfer from Guh et al. (2008) Each case of diarrhea avoided valued at HTG 715	Alternative 1: Monetized YLD C third-party costs Each case of diarrhea avoided valued at HTG 306
3%	HTG 709 USD 11	HTG 317 USD 5
5%	HTG 707 USD 11	HTG 316 USD 5
12%	HTG 700 USD 11	HTG 311 USD 5

Source: Estimates by the authors. All values are in millions of HTG or millions of USD.

The results indicate that in the base case the value of avoided non-fatal health risks is HTG 707 million (USD 11 million) per year. The alternative approach of using monetized YLD + third-party costs leads to a lower value of non-fatal health risks, HTG 309 million (USD 5 million) per year or only 44% of the base case.

### 5.5 Lifetime productivity benefits

The results of Section 4 indicate that there is a 20% reduction in stunting from the intervention, avoiding 55,000 cases of stunting per year at steady state. It is typical to assess the long-term benefits of reduced stunting as an increase in lifetime productivity (Hoddinott et al., 2013*a*; Horton & Hoddinott, 2014).

Due to the long period of time that must elapse between provision of nutrition in childhood and identifying effects in adulthood, only two studies were identified that have attempted to estimate the long-term consequences of not being stunted on lifetime productivity.^16^ The seminal studies in this genre are by Hoddinott et al. (2008) and Hoddinott et al. (2011). Both studies concern a group of Guatemalan children who were provided with protein supplementation (*atole*) in 1969–1977 and were tracked down and identified in 2002–2004. Compared to a control group that were provided with no protein (*fresco*), the treatment cohort had 25 percentage points (45% versus 20%) lower prevalence of *severe* stunting. The 2008 follow-up study indicates that at that time men exposed to *atole* had 46% higher hourly wages than those exposed to *fresco*, while there was no difference in wages for women. The 2011 study shows that men have 20% higher hourly earnings for each 1 S.D. increase in height for age z-scores, while for women the increase is 7.2% but it is not statistically significant. The authors also estimate that those who were stunted as children had 66% lower per capita *consumption* 35 years later compared to their non-stunted peers. These effects appear to be driven by increases in cognitive ability and human capital associated with avoided stunting. For example, those that were not stunted had a 28-percentage-point greater likelihood of being employed in high skilled or white-collar profession, and were more likely to own their own businesses.

Victora et al. (2008) summarize the available findings (at that time) from longitudinal cohort studies in Brazil (Victora et al., 2003), Guatemala (Grajeda et al., 2005; Martorell et al., 2005) and India (Bhargava et al., 2004; Sachdev et al., 2005) of childhood height for age z-score on future income and assets. They note that a 1-point height for age z-score increase at age two is associated with 8% higher wages in Brazil, 8–25% higher wages in Guatemala and 18–27% more assets in India at adulthood. It is important to clarify that these longitudinal, observational studies are inherently less suitable for estimating causal effects of stunting on wages than the randomized controlled trial conducted in Guatemala. McGovern et al. (2017) state that the median effect across a range studies that control for unobserved confounding and measurement error is that a 1 cm increase in height is associated with a 4% and 6% increase in wages for men and women, respectively.

There is evidence that stunting reduces earning capacity and consumption. The only long-term experimental study available suggests that exposure to an intervention that has a significant effect on stunting is responsible for an average boost to wages of 46% in Guatemala, a figure we apply in this study. It is possible that the intent-to-treat coefficient is an underestimate of the impact of stunting on wages, since it accounts for the fact that some children that received the nutritional supplement may not have consumed the supplement. At the same time, the effect was only noted for men, and not for women, but in this study, we apply the boost to the entire population which may suggest that 46% would be too high an estimate. In any case, this assumption is more conservative than previous benefit-cost analyses that have adopted a boost of 59% to the entire population who avoid stunting (Hoddinott et al., 2013*a*; Horton & Hoddinott, 2014). In sensitivity analyses we explore the effects of varying this assumption on the results.

Key assumptions of the calculation:

- avoiding stunting boosts wages by 46% in adulthood;
- the intervention results in increased wages in adulthood beginning at age 16 until 60;
- the average wage of a Haitian adult in 2016 is HTG 51,505 (USD 815) and is assumed to grow in accordance with GNI per capita growth assumptions. The wage rate is calculated by the equation, wage = GNI per capita ∗ labor force participation / labor share of income. GNI per capita is HTG 44,891 (USD 710) (World Bank, 2018), labor force participation rate is 43.5% and the labor share of income is assumed to be 50%.

The results of this calculation are presented in Table 8.

**Table 8:** Annualized lifetime productivity benefit.

Discount rate	Benefit
3%	HTG 23,025 USD 364
5%	HTG 12,743 USD 201
12%	HTG 2,525 USD 40

Source: Estimates by the authors. All values are in millions of HTG or millions of USD.

Clearly the discount rate has a pronounced effect on lifetime productivity benefits. This is unsurprising given the long-lived nature of the benefits (up to 60 years after the intervention) and the fact the benefits of increased productivity only start 16 years after the intervention commences. Even moving from a 3% to 5% rate would almost halve the benefits. Choosing a 12% discount rate diminishes the benefits significantly and shifts the relative importance of the intervention firmly toward avoided mortality and non-fatal health risks.

## 6 Discussion and conclusion

Table 9 summarizes the results of the previous sections using base case estimates. The base case includes avoided mortality benefits using a VSL of HTG 1.3 million (USD 20,500), non-fatal health effects using benefit transfer from Guh et al. (2008) and productivity benefits. The provision of the early child nutrition package is beneficial in the Haitian context.

**Table 9:** Summary of costs and benefits of nutrition intervention – base case results (HTG millions).

	Annualized benefit				Annualized Cost	Summary measures	
Discount	Mortality avoided	Non-fatal health risks avoided	Productivity	Total	Total	Benefit to Cost Ratio	Net benefit per year
3%	3,129	709	23,025	26,593	3,182	8.4	23,680
5%	3,101	707	12,743	16,294	3,178	5.2	13,372
12%	3,009	700	2,525	6,019	3,166	2.0	3,068

If the intervention package can be scaled up to 90% of the target population, it is expected to annually prevent about 55,000 cases of child stunting, 7,600 babies born with low birthweight and 28,000 cases of maternal anemia. These nutrition improvements will avoid 1,830 under-five deaths, 80 maternal deaths and 900,000 episodes of child illness every year. For those children who avoid being stunted, it will deliver productivity benefits equivalent to 5× GNI per capita in present value terms, at a 5% discount rate.

The benefits of the intervention will favor poorer sections of Haitian society since nutrition and health outcomes tend to be negatively correlated with wealth (IHE and ICF, 2016). For example, households in the lowest quintile of wealth are 3.5 times more likely to have stunted children, half as likely to have fully vaccinated children and 40%–50% less likely to seek health treatment for diarrhea, fever or respiratory infection.

However, the costs of the intervention are not small at HTG 16,222 (USD 248) per child over two years. The marginal costs required to scale up the package of interventions to 90% are HTG 3.2 billion (USD 50 million) per year. The BCR is 5.2 at a 5% discount rate. Other studies focusing on health, nutrition and education interventions in the *Haiti Priorise* series suggest there are more effective ways to use a marginal gourde to build an improved human capital base in Haiti (Lomborg et al., 2018). For example, wheat flour fortification which has a centrally estimated BCR of 24 (Engle-Stone et al., 2017) and early childhood stimulation with a BCR of 17 (Rabbani, 2017).

To test the strength of this conclusion, we address uncertainty in parameter estimates by conducting a Monte Carlo probability simulation. The analysis varies numerous parameters in the model including the LiST model impacts for each year (stunting, under-five mortality, under-five non-fatal health effects, maternal mortality), the VSL, individual WTP to avoid diarrhea, the productivity gains from reduced stunting, the short-term (year 0–5), medium-term (year 6–26), and long-term (year 27–73) GNI per capita growth, and the costs of the intervention.

To run the Monte Carlo simulation, we have to make assumptions regarding the distribution of the parameters. We assume the health parameters (stunting, under-five mortality, under-five non-fatal health effects, maternal mortality) are normally distributed. The mean values of the normal distributions are then estimated using the base case as presented by LiST. The standard deviation for each normal distribution was estimated by averaging half the difference between the mean and lower and upper bounds, which represent the 95% confidence intervals obtained from LiST (see Section 4). We assume the other parameters were distributed uniformly due to limited data.

For the VSL, Robinson et al. (2019) propose a minimum value of 20× GNI capita and a maximum value of 160× GNI per capita, which we use as the lower and upper limits of the uniform distribution. The minimum of 20× GNI capita^17^ represents a theoretical floor proposed by Robinson et al. (2019) while the maximum, 160× GNI per capita is the largest value suggested by the same paper. The range of the WTP to avoid diarrhea and productivity gains from avoided stunting represent the ends of the range of effect sizes documented in Guh et al. (2008) and Hoddinott et al. (2008) respectively. Growth rates span +*/*− 100% of the average of the corresponding base case, while unit costs span +*/*− 20%. We present the varied parameters and their ranges in Table 10.

**Table 10:** Summary of parameter estimates for Monte Carlo simulation of costs and benefits of nutrition intervention.

Parameter	Minimum	Maximum
VSL	20^*^ GNI per Capita	160^*^ GNI per Capita
Willingness to pay to avoid diarrhea	1% of GNI per Capita	2.2% of GNI per Capita
Productivity gains from avoided stunting	11%	82%
Short-term GNI per capita growth	0%	5%
Medium-term GNI per capita growth	0%	3%
Long-term GNI per capita growth	0%	1.2%
Cost of Intervention per child	HTG 13,000 USD 205	HTG 19,500 USD 310
	**Mean**+	**Standard Deviation**+
Nutrition and health impacts (stunting, under-five mortality, under-five non-fatal health effects, maternal mortality)+	Base case from LiST	Average of the half the difference between the Low and High end of 95% C.I. from LiST

+ The nutrition and health impacts are assumed to have a normal distribution so we present the mean and standard deviation in Table 10 above.

In the Monte Carlo simulation the LiST impact parameters vary each year but are associated with the previous year’s LiST impacts. We associate parameters across years because we assume that an underperforming (overperforming) intervention in one year will likely also underperform (overperform) in the subsequent year. For example, we assume that stunting in year 2 has a 0.7 association with the stunting in year 1.^18^ We make this same assumption for each year for maternal mortality, cases of diarrhea, and under-five mortality. Additionally, we fix the discount rate at two times the average short-term growth rate. Therefore, since we assume the short-term growth varies from 0% to 5%, the discount rate varies from 0% to 10%. Otherwise, no other parameters are associated with one another.

**Figure 1:**
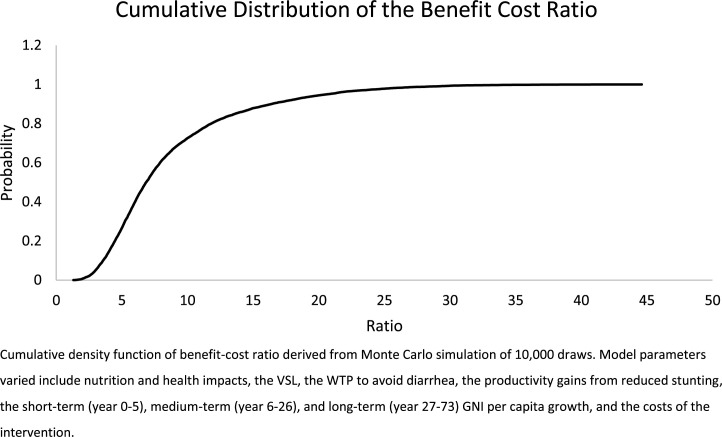
Cumulative distribution of results from a Monte Carlo simulation (10,000 draws) of a package of early childhood interventions to improve nutrition in Haiti (Benefit-Cost Ratio).

The results of the Monte Carlo analysis with 10,000 trials show the project had a BCR greater than one in every draw. The cumulative density function (CDF) of the BCRs is presented in Figure 1 (the same figure for annualized net benefits is presented in the appendix). The BCR ranges from 1.3 to 44.6 with a mean of 8.6 and a median of 6.8. In every simulation the project passes a cost-benefit test, and in some simulations the project is extremely beneficial. However, 91% of the draws have a BCR less than 17, the central estimate for early childhood stimulation and 97% of the draws have a BCR less than 24, the central estimate for wheat flour fortification. This supports the conclusion that this package of interventions may not be the most efficient use of a marginal gourde in Haiti.

**Figure 2:**
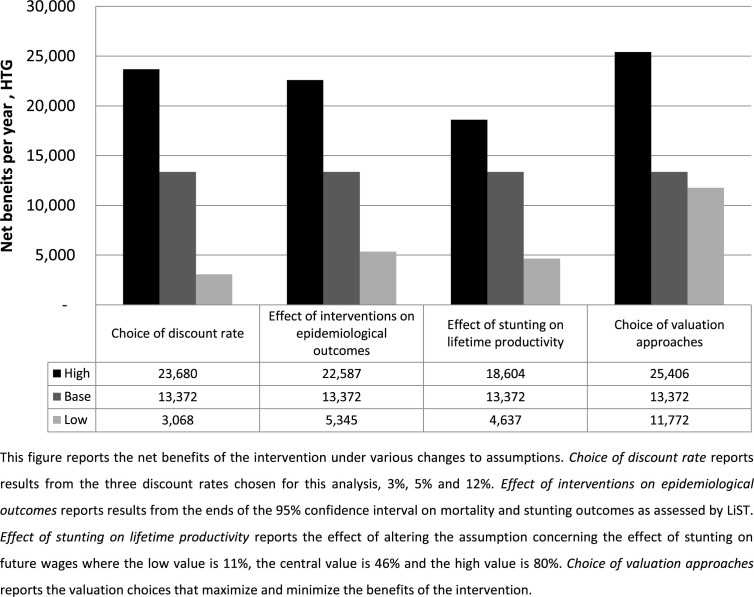
Effect of uncertainty on net benefits.

Last, we explore how uncertainty in these methods might affect decision makers using this technique to make choices that maximize expected welfare. To assess this, we present the combination of valuation choices that maximize net benefits against the choices that minimize net benefits,^19^ and compare these to the variation derived from other important sources of uncertainty: discount rates, nutrition and health impacts, and productivity gains from avoided stunting. We use the same minima and maxima presented in Table 10 and for discounting we use 3% and 12%. The results of these tests are presented in Figure 2 and compared to the base case estimate.

The results indicate that, in this particular study, uncertainty in valuation methods creates slightly smaller variation in the net benefits and BCR estimates than other sources of uncertainty. The estimated net benefits from various discount rates range from a lower bound of HTG 3 billion (BCR = 2.0) to an upper bound estimate of HTG 24 billion (BCR = 8.5). The inherent uncertainty in the intervention’s effect on mortality and nutrition outcomes results in a range of net benefits from as low as HTG 5.3 billion (BCR = 2.7) to a high of HTG 22.6 million (BCR = 8.1). The uncertainty in the effects of stunting on lifetime productivity results in a range of benefits from as low as HTG 4.6 billion (BCR = 2.5) to a high of HTG 18.6 billion (BCR = 6.9). The choice of valuation methods results in a range of estimates from as low as HTG 11.7 billion (BCR = 4.7) to as high as HTG 25.4 billion (BCR = 9.0).

Based on this analysis, it would seem there is insufficient precision in the current state of knowledge to confidently discern between interventions with BCRs ranging from 2 to 9. While this uncertainty might appear large, reviews of BCRs or cost-effectiveness ratios have demonstrated that the span of the distribution across interventions in a given policy space can be much larger, with the difference between a typical and an outlier intervention being 2–4 orders of magnitude apart. For example, Ord (2013), citing Jamison et al. (2006) demonstrates that within global health the cost-effectiveness of all interventions covered in Disease Control Priorities 2 span a range of four orders of magnitude from 0.02 to 300 DALYS per $1000, while the median value is 5. In a review of primary school interventions in the developing world, McEwan (2015) shows that the cost to improve test scores by 0.2 standard deviations has a range of less than USD 1 to more than USD 1000 across 76 randomized controlled trials, with a median around USD 20. Therefore, despite the uncertainty created by valuation methods (+*/*−50%), it is unlikely to overwhelm credibly identified outliers of effectiveness. This is an important feature given that the optimal strategy of a social welfare maximizing decision maker is to identify and implement outliers of effectiveness (Ord, 2013).^20^ This suggests that benefit-cost analysis is useful for such an exercise even with the gaps in knowledge documented in Robinson et al. (2019) and associated papers.

## Figures and Tables

**Figure 3:**
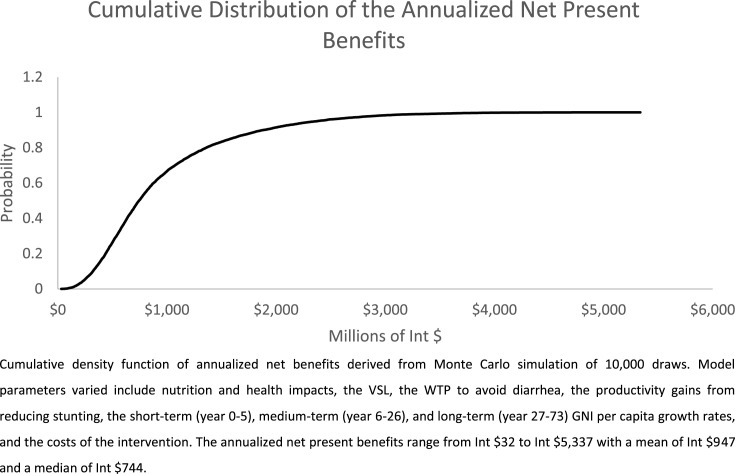
Cumulative distribution of results from a Monte Carlo simulation (10,000 draws) of a package of early childhood interventions to improve nutrition in Haiti (Net Benefits).

## Footnotes

2 This package of interventions, or variations of it, has been ranked highly in three previous Copenhagen Consensus exercises (Horton et al., 2009; Hoddinott et al., 2013*a*; Horton & Hoddinott, 2014).

3 Additionally, as defined in this paper, mortality benefits solely capture the change in the risk of premature mortality, while non-fatal health risks include illness that might occur prior to death (as well as illness that does not pose a risk of mortality).

4 Lifetime productivity benefits from avoided stunting start at age 16, extend throughout adulthood and are assumed to be a function of a nation’s income. Therefore, in countries with a greater prevalence of stunting and higher projected growth rates (e.g. India), the share of productivity benefits will likely be higher.

5 Unless otherwise stated, all currencies are in 2016 figures. Throughout this study, the following 2016 exchange rates are used: USD 1 = HTG 63.34 and Int$1 = HTG 25.03 (World Bank, 2018).

6 As discussed in Hammitt (2017) and Robinson et al. (2019), individuals are likely to consider the loss in future earnings when valuing reductions in their mortality risks. However, they are likely to only consider what they expect to earn under baseline conditions, which includes the risk of stunting. Decreasing stunting likely increases earnings beyond what would otherwise be expected, due to a combination of improved cognitive ability, physical stature and education that accompany avoided stunting (Prendergast & Humphrey, 2014).

7 Most notably, the interventions in this package form the basis of *Scaling Up Nutrition*, a multilateral movement backed by significant government funding committed to ending undernutrition across the world (Scaling Up Nutrition, 2014).

8 While the project itself will only be implemented from 2016 to 2025, we require the long-term growth rates to calculate the lifetime productivity benefits attributable to the intervention.

9 *Haiti Priorise* was a research and advocacy project conducted by the Copenhagen Consensus Center over the period 2015 to 2017. The project commissioned 85 cost-benefit analyses across 18 topic areas covering key Haitian policy questions. For more information see www.haitipriorise.com.

10 For the purposes of this exercise, we take Bhutta et al.’s AFRO D region costs to be the relevant comparator for Haiti, which are presented in the appendix of that paper on p 31.

11 This calculation spreads the high cost of treatment over a wider universe of children who will receive the full preventative regime, recognizing that only a relatively small proportion of children will require treatment.

12 The VSL does not represent the value of avoiding certain death. Rather it reflects the WTP for small reductions in mortality risk.

13 Robinson and Hammitt (2018) recommend that averted costs be used as a proxy when WTP estimates are not available, including medical and productivity costs that would otherwise be borne by the individual as well as third parties. They recommend that monetized YLDs be used in sensitivity analysis. In this study, we do not apply the cost-of-illness approach since it undervalues avoided morbidity by excluding non-monetary costs such as inherent pain and suffering.

14 Guh et al. (2008), Table 4 reports WTP figures for 0–1 year olds and 2–5 year olds separately. Therefore, it is possible to assign different valuations for these different age groups. However, given the small sample size of the 0–1-year-old group (12), we combine the results for the two groups to estimate WTP for avoiding diarrhea in children under 5 generally.

15 The monetized YLD component of this value increases proportionally with VSLY growth (see Section 5.3) while the third-party costs increase proportionally with assumed GNI per capita growth.

16 See McGovern et al. (2017) for a recent, thorough review of the evidence.

17 This theoretical floor is intended to represent future lifetime earnings, assuming that life expectancy is at least 20 years and that the VSL does not fall below future earnings.

18 We assume for all health impacts (stunting, under-five mortality, under-five diarrhea morbidity, and maternal mortality) that number of cases avoided each year has a 0.7 association with the number of cases avoided in the previous year.

19 Since the base case VSL documented in Section 5.3 is already the lowest VSL value available, for the purposes of this sensitivity analysis we use a low VSL representing 20× GNI per capita, consistent with Table 10.

20 As an example, if the typical intervention in a portfolio has say, a BCR of 5 and the most effective intervention possible is 500, doubling the efficiency of 99% of the funds under control (an extremely difficult endeavor even under perfect certainty) would deliver as much social welfare as directing 1% of the funds to the most effective intervention available.

## Appendix A

### A.1 Assumptions used to calculate non-fatal health benefits

The calculation of outpatient costs assumes:

- 32% of children with diarrhea are taken to health care facilities for treatment based on estimates from the 2016–17 demographic and health survey (IHE and ICF, 2017).
- Each outpatient visit in Haiti costs HTG 487 or USD 7.50 (McBain et al., 2017).
- 65% of all costs are borne by public or NGO health facilities (World Development indicators, World Bank 2016).

The calculation of inpatient costs assumes:

- 8.2% of patients who seek care are referred to a hospital facility (Hutton et al., 2007).
- Each outpatient visit in Haiti costs HTG 3900 or USD 60.00 (Sklar, 2017).
- 65% of all costs are borne by public or NGO health facilities (World Bank 2016).

The calculation of caregiver costs assumes:

- One adult is responsible for caregiving across the entire duration of the illness.
- The average, unskilled rural wage is HTG 2600 per month.
- Cost of time is approximated by 50% of rural wage as per Whittington and Cook (2019).
- The average duration of an episode of diarrhea is calculated at 6.0 days (GBD, 2016).
